# Subperiodic groups, line groups and their applications

**DOI:** 10.1107/S1600576724003418

**Published:** 2024-05-31

**Authors:** Gemma de la Flor, Ivanka Milošević

**Affiliations:** a Institute of Applied Geosciences, Karlsruhe Institute of Technology, Karlsruhe, Germany; b Faculty of Physics, University of Belgrade, Studentski trg 12, 11001 Belgrade, Serbia; The University of Western Australia, Australia

**Keywords:** layer groups, rod groups, line groups, symmetry, nanostructures

## Abstract

Understanding the symmetries described by subperiodic groups – frieze, rod and layer groups – has been instrumental in predicting various properties (band structures, optical absorption, Raman spectra, diffraction patterns, topological properties *etc*.) of ‘low-dimensional’ crystals. This knowledge is crucial in the tailored design of materials for specific applications across electronics, photonics and materials engineering. However, there are materials that have the property of being periodic only in one direction and whose symmetry cannot be described by the subperiodic rod groups. Describing the symmetry of these materials necessitates the application of line group theory. This paper gives an overview of subperiodic groups while briefly introducing line groups in order to acquaint the crystallographic community with these symmetries and direct them to pertinent literature. Since line groups are generally not sub­periodic, they have thus far remained outside the realm of symmetries traditionally considered in crystallography, although there are numerous ‘one-dimensional’ crystals (*i.e.* monoperiodic structures) possessing line group symmetry.

## Abstract 2

Since they do not belong to the class of subperiodic groups, line groups thus far have been beyond the scope of symmetries traditionally studied in crystallography, although they describe the symmetry of numerous ‘one-dimensional’ crystals (*i.e.* monoperiodic structures). Therefore, together with an overview of frieze, rod and layer groups, a brief introduction to line groups is presented.

## 1. Introduction

In the same way in which three-dimensional space groups are used to describe the atomic structure of three-dimensional crystals, subperiodic groups are used to describe the atomic structure of other crystalline structures, such as liquid crystals, domain interfaces, twins, thin films, layered materials, polymers, materials with pronounced rod arrangements, nanotubes and nanowires. The interest in materials with subperiodic symmetry is constantly growing due to their scientific and technological relevance. There are 80 layer groups (three-dimensional groups with two-dimensional translations) which, together with the 75 rod groups (three-dimensional groups with one-dimensional translations) and the seven frieze groups (two-dimensional groups with one-dimensional translations), constitute the subperiodic groups. The crystallographic data for subperiodic groups are compiled in *International Tables for Crystallography*, Vol. E, *Subperiodic Groups* (Kopský & Litvin, 2010 ▸; henceforth abbreviated as *IT* E), and also freely available online in the section of the Bilbao Crystallographic Server (https://www.cryst.ehu.es/) (Aroyo *et al.*, 2011 ▸; Tasci *et al.*, 2012 ▸) dedicated to the subperiodic groups.

In crystallography, two-dimensional sections of crystal structures are fundamental in bicrystallography, for the study of bicrystals. The terms bicrystallography and bicrystals were first introduced by Pond & Bollmann (1979 ▸) in the study of grain domains. In general, a bicrystal is defined as two crystals, usually with the same structure but different orientation with a common boundary or interface. The identification of the layer group of a section is very useful in bicrystals to describe both the bicrystal itself and the symmetry of the boundary plane. In the case of domain walls and twin boundaries, which can be considered a special case of bicrystals, there are crystallo­graphic restrictions on their orientations. The layer symmetries of the sections of the 230 space groups are collected in the ‘scanning tables’ in *IT* E. In addition, it is possible to calculate the layer symmetry of a periodic section using the program *SECTIONS* in the Bilbao Crystallographic Server. Layer groups are useful to describe the symmetry of layered materials with scientific and technological relevance such as graphene, black phosphorus, hexagonal boron nitride and transition metal dichalcogenides. The symmetry of domain twins and domain walls, as well as the symmetry of woven textiles (Hammond, 2009 ▸; De Las Peñas *et al.*, 2024 ▸), can also be described by layer groups. Rod groups, on the other hand, can be used to study crystals with pronounced rod arrangements, such as benitoite (BaTiSi_3_O_9_), garnet or catapleite (Na_2_ZrSi_3_O_9_·2H_2_O). The identification of the rod symmetry of a straight line penetrating the crystal, called the penetration rod group, is also very useful to study systems with periodicity in a single direction such as line dislocations or intersections of boundaries. The penetration rod groups of space groups are not compiled in *IT* E; to the best of our knowledge this information can only be calculated using the program *RODS* in the Bilbao Crystallographic Server.

There are other three-dimensional systems with periodicity in a single direction which cannot be described by any of the 75 rod groups tabulated in *IT* E. This is the case of the mono-periodic compounds that are invariant under the rotations of noncrystallographic order. Their full symmetry is given by line groups (Damnjanović & Milošević, 2010 ▸). Examples include certain polymers like DNA double helix (Milošević *et al.*, 1996 ▸), carbon (Damnjanović *et al.*, 1999 ▸) and transition metal dichalcogenide nanotubes (Milošević *et al.*, 2000 ▸), ZnO nanosprings (Milošević *et al.*, 2006 ▸), structural analogs of single-wall carbon nanotubes (De Las Peñas *et al.*, 2014 ▸; Loyola *et al.*, 2012 ▸) *etc*. Line groups also describe the symmetry of systems having only helical periodicity: the vast majority of multi-wall (Damnjanović *et al.*, 2003 ▸) and helically coiled carbon nanotubes (Milošević *et al.*, 2012 ▸), extended metal atom chains (Ou & Jin, 2018 ▸), nanohelicenes (Domnin *et al.*, 2023 ▸), polytwistanes (Domnin *et al.*, 2022 ▸), helical polyacetylenes (Porsev & Evarestov, 2022 ▸), and incommensurately modulated crystals (Mészáros *et al.*, 2020 ▸).

The use of line groups is essential for efficient computation of the physical properties of quasi-one-dimensional (hereafter referred to as quasi-1D) systems and for achieving a complete and clear interpretation of the obtained results. A seminal paper on the full symmetry of carbon nanotubes (Damnjanović *et al.*, 1999 ▸) revealed that their symmetry was described by nonsymmorphic line groups, aiding comprehension and correct interpretation of carbon nanotube (CNT) Raman spectra (Reich *et al.*, 2004 ▸). In addition, it highlighted the minimal interaction between the walls in multi-wall CNTs and, on the basis of the symmetry arguments only, deduced the presence of the Goldstone-mode-like super-slippery sliding of the incommensurate walls of multi-wall CNTs (Damnjanović *et al.*, 2002 ▸).

Generally, a wide range of applications employing line-group-symmetry-based methods emerged in the investigation of quasi-1D systems (Damnjanović *et al.*, 2005 ▸). The quantum-mechanical methods based on line group and layer group symmetry are implemented in the program *POLSym* (Damnjanović & Milošević, 2015 ▸), a platform for numerical computation which enables one to estimate the stability of low-dimensional structures, to calculate dispersion relations for electrons, phonons and magnons, to generate diffraction patterns and optical and Raman spectra, *etc*. Furthermore, *POLSym* has recently been upgraded to incorporate the tools for topological characterization of nonmagnetic low-dimensional matter by means of elementary band (co)-representations for single and double (gray) line groups (Milošević *et al.*, 2020 ▸; Dmitrović *et al.*, 2022 ▸).

The main aim of this paper is to indicate the primary differences between subperiodic and line groups, and introduce the line groups and their applications in crystallography for describing systems that cannot be described by subperiodic crystallographic groups, the rod groups.

## 2. Subperiodic groups: frieze, rod and layer groups

The crystallographic subperiodic groups are classified into frieze, rod and layer groups. Layer and rod groups are three-dimensional groups with, respectively, two- and one-dimensional translations, and frieze groups are two-dimensional groups with one-dimensional translations. This classification underlines the existing relationship between subperiodic and space groups, *i.e.* a group–subgroup relation exists between the subperiodic groups **S** and the space groups **G**. Each layer and rod group corresponds to a three-dimensional space group, while each frieze group is associated with a two-dimensional space group (called the plane groups). These relationships have been considered in detail in the literature [see *e.g.* Wood (1964 ▸), *IT* E and references herein]. The space group **G** to which a subperiodic group **S** is related can be expressed as a semi-direct product of **S** with a one- or two-dimensional translation group **T** ^(*i*)^ of additional translations, such as 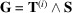 , where **T** ^(*i*)^ is a normal subgroup of **G**. This means that each subperiodic group **S** is isomorphic to the factor group **G**/**T** ^(*i*)^ (Litvin & Kopsky, 1987 ▸, 2000 ▸). For example, the layer group *pmmm* (No. 37) is a subgroup of the three-dimensional space group *Pmmm* (No. 47); this means that the layer group *pmmm* is isomorphic to the factor group *Pmmm*/**T** ^(3)^, where **T** ^(3)^ is the translational subgroup of all translations along the third (*z*) direction. The layer group *pmmm* is the symmetry of the plane, transecting a crystal of three-dimensional space group symmetry *Pmmm*, perpendicular to the *z* axis, at *z* = 0.

The isomorphism between **S** and the factor group **G**/**T** ^(*i*)^ results in a close relationship between the Wyckoff positions, maximal subgroups, minimal supergroups, Brillouin zones and irreducible representations (irreps) of **S** and **G**. One can show, for example, that the set of Wyckoff positions of a layer group can be directly derived from those of the related space group, *i.e.* the set of Wyckoff positions of a layer group is contained in the set of Wyckoff positions of the related space group (*cf.* Evarestov & Smirnov, 1993 ▸). The restrictions imposed by the loss of periodicity in the third (*z*) direction result in the following restrictions on the special-position coordinates of layer groups: only the special positions of **G** whose *z* coordinate does not involve a fraction of the unit-cell parameter are possible special positions of the layer group, *i.e.* special positions of the space group with *z* coordinates (*z*, −*z* or 0). In that way, to each Wyckoff position of the layer group there corresponds exactly one Wyckoff position of the corresponding space group, specified by exactly the same site-symmetry group and multiplicity, and by the same set of coordinate triplets of equivalent positions. In accordance with the conventions adopted in *IT* E, the labeling of the Wyckoff position letter of layer groups is done independently of that of space groups. This means that the Wyckoff letters of the corresponding space and layer group Wyckoff positions might not coincide, in general. On the other hand, the irreps of layer groups, established and derived by Litvin & Wike (1991 ▸) and Milošević *et al.* (1998 ▸), can also be derived from those of the corresponding space group due to the existing isomorphism between **S** and **G**/**T** ^(*i*)^. Note that all the irreps of a layer group are contained in the irreps of the related space group **G**, and every irrep of **S** is related to a specific irrep of **G** (Evarestov & Smirnov, 1993 ▸; Smirnov & Tronc, 2006 ▸). Similarly, the Brillouin zones of layer groups were derived from those of space groups by de la Flor *et al.* (2021 ▸).

As a practical example of the use of subperiodic groups, the symmetry of layered and rod materials is analyzed.

### 2.1. Materials with layer symmetry: WS_2_ and MoS_2_

The arrangement of atoms in a layer cannot be adequately characterized by any of the 17 two-dimensional or any of the 230 three-dimensional space groups. These atom arrangements are characterized by a two-dimensional translational periodicity and a finite thickness in the third dimension. This thickness allows possible three-dimensional reflections, glide planes and twofold screw axes not included in two-dimensional space group symmetry. Therefore, the symmetry group of such an arrangement of atoms has to be described with one of the 80 layer groups and not with a plane group. Note that the absence of periodicity in the third dimension within layer groups results in the exclusion of specific symmetry elements: (i) screw axes normal to the plane of periodicity; (ii) glide planes with glide directions out of this plane; and (iii) *n*-fold axes not normal to this plane, with *n* > 2.

In layered crystals the interaction between nearest layers is usually weaker than that between nearest atoms in the same layer plane. Depending on the interaction between the layer and the bulk, these sorts of materials with layer symmetry can be classified into five different types: (i) pure layered systems like free-standing films; (ii) single layers in layered crystals which can be separated from the bulk due to a weak van der Waals interlayer interaction; (iii) artificial nanolayers grown on substrates or between two bulk materials where the interaction between the nanolayer and surrounding bulk materials is neglected; (iv) layers (or slabs) which model atomically clean crystal surfaces where the slab interaction with the rest of the semi-infinite crystal is neglected; (v) interfaces between different crystals, including domain walls approximated by atomically clean crystal surfaces.

**Figure 1:**
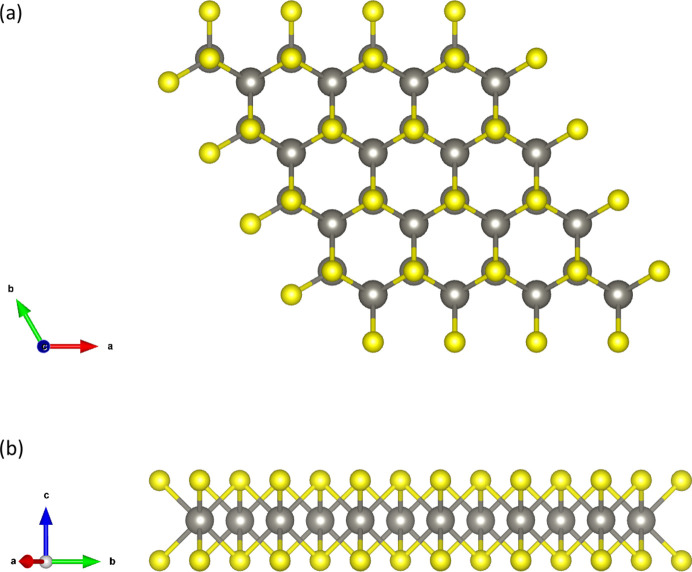
(*a*) The projection of the crystal structure of WS_2_ (MoS_2_) along [001], and (*b*) the crystal structure of a single layer of WS_2_ (MoS_2_).

As an example, let us consider the transition metal dichalcogenides WS_2_ and MoS_2_. In layered and multilayered materials, the symmetry of the bulk is described by a space group, while the symmetry of the layers is described by layer groups. The space group of the WS_2_ and MoS_2_ bulk crystals is *P*6_3_/*mmc* (No. 194). In the bulk crystal, the metal atoms occupy the 2*c* (1/3; 2/3; 1/4) position and the sulfur atoms occupy the 4*f* (1/3; 2/3; *z*) position (Lee *et al.*, 2014 ▸). The layer group of a single layer is 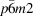 (No. 78) and is isomorphic with the factor group **G**/**T** ^(3)^, where **G** is the space group 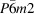 (No. 187) and **T** ^(3)^ is the one-dimensional translation group along the third (*z*) direction, *i.e.* 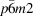 is a subgroup of 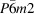 . The crystal structure of a single layer is shown in Fig. 1 ▸. Atoms in the primitive unit cell of the layer occupy the following Wyckoff positions: W(Mo) 1*c* (2/3; 1/3; 0) and S 2*e* (1/3; 2/3; *z*). Note that the layer group of a single layer is also a subgroup of the space group of the bulk crystal.

### 2.2. Materials with rod symmetry: α-Se (or α-Te) and SnIP

**Figure 2:**
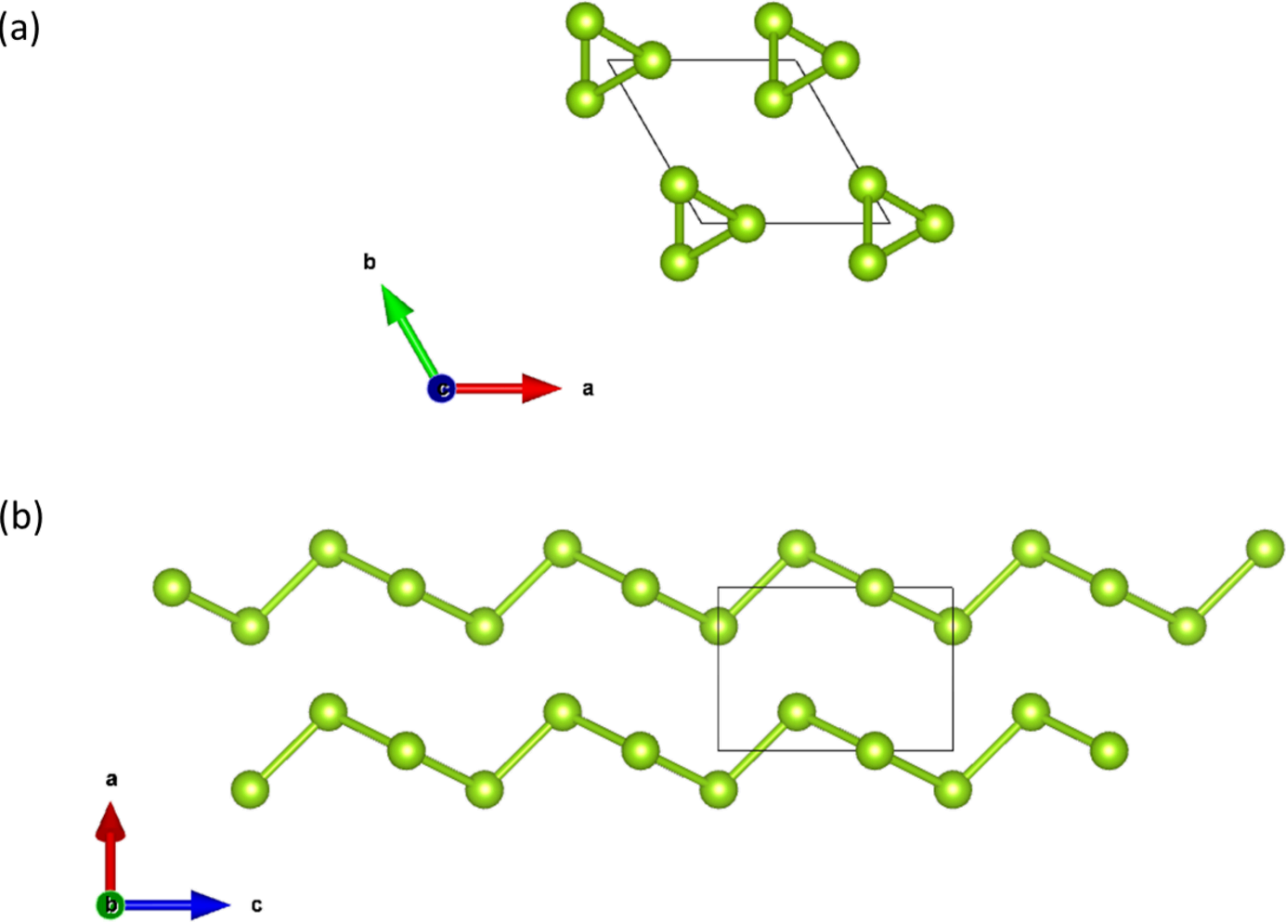
(*a*) The projection of the crystal structure of α-Se (α-Te) along [001], and (*b*) the spiral chains along the *c* direction of the crystal structure of α-Se (α-Te).

**Figure 3:**
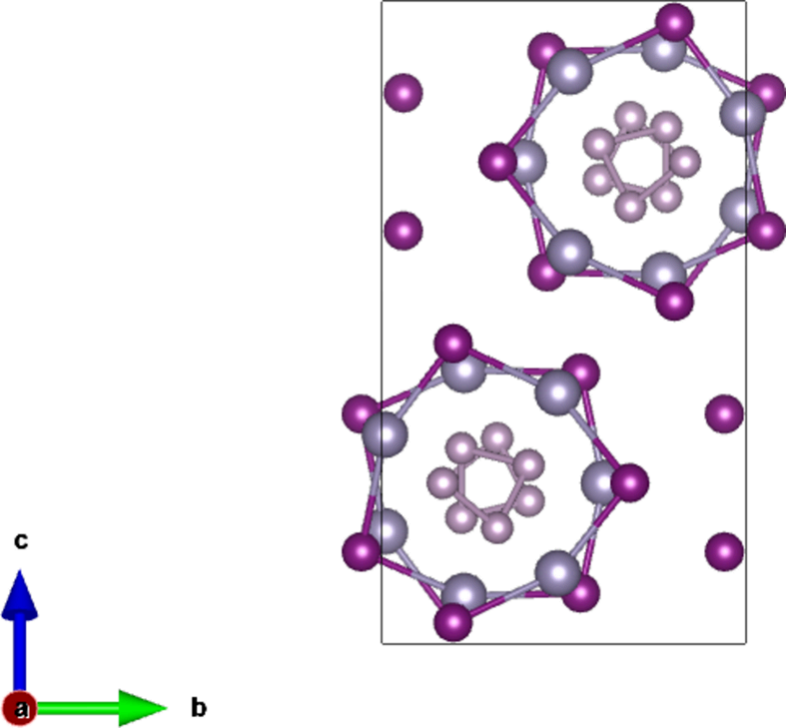
The projection of the crystal structure of SnIP along [100]; the spiral chains are along this direction.

As an example of materials with rod symmetry, the structures of α-Se and α-Te (see Fig. 2 ▸) and SnIP (see Fig. 3 ▸) were chosen. These are three-dimensional structures constituted of infinite chains parallel to a certain direction. In such materials, the symmetry of the bulk is described by a space group, while the symmetry of the individual chains, in certain cases, can be characterized by one of the 75 crystallographic rod groups, although this is not always the case.

The stable form at atmospheric pressure of α-Se and α-Te crystallizes in the trigonal space group *P*3_1_21 (No. 152) occupying the Wyckoff position 3*a* (*x*, 0, 1/3). McCann & Cartz (1972 ▸) and Bradley (1924 ▸) reported *x* = 0.227 and *x* = 0.269 for Se and Te atoms, respectively. A characteristic of these structures is the infinite helical chains aligned parallel to the *c* axis, *i.e.* the atoms of Se (or Te) are arranged in infinite spiral chains along the *c* axis of the structure (see Fig. 2 ▸). The atoms along the chain form covalent bonds while metallic and van der Waals forces hold the chain together. In this particular case, the symmetry of the spiral chain of Se or Te atoms can be described by one crystallographic rod group, *i.e.* the rod group 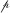 3_1_21 (No. 47).

On the other hand, the structure of SnIP crystallizes in the monoclinic space group *P*2/*c* (No. 13) (Pfister *et al.*, 2016 ▸). The structure of this compound is constituted by two substructures, (i) a helical chain of P atoms and (ii) a second helical chain of Sn and I atoms, which form double helices parallel to the [100] direction (see Fig. 3 ▸). The symmetry of these helical chains cannot be described with one of the 75 crystallographic rod groups, since the helix has a 7_4_ screw axis, if the helix is right handed, or 7_3_, if it is left handed. In the literature, depending on whether the helix is right or left handed, the symmetry of the helical chain is described by the noncrystallographic rod group 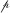 7_4_21 or 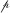 7_3_21 (following the Hermann–Mauguin notation). The symmetry of the helical chain can also be described by the line groups **L**7_4_2 and **L**7_3_2, respectively.

## 3. Line groups

In general, systems that exhibit periodicity in only one direction can be described by line groups (hereafter referred to as LGs). The structure of these groups can be understood as a combination of the intrinsic symmetry of individual building blocks, monomers, and a group of generalized translations. These translations arrange the monomers along a single direction of periodicity (the *z* axis, by convention). The LG **L** represents the geometrical symmetries of a polymer (*e.g.* molecular chains, nanotubes, nanosprings) and includes an infinite cyclic subgroup of generalized translations **Z** generated by an element *z* = {*R* ∣ *f*}, where *f* is an elementary (fractional) translation along the *z* axis and *R* is an orthogonal transformation which preserves the orientation of the *z* axis, *i.e.* a rotation around the *z* axis or a reflection σ_*v*_ containing the *z* axis. In the former case, the helical or screw-axis group **T**_*Q*_(*f*) is generated by a rotation of 2π/*Q* around the *z* axis (where *Q* can be any real number), followed by a fractional translation *f* along it, while in the latter case, the glide-plane group **T**′ is generated by the reflection σ_*v*_ followed by the translation 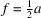 , where *a* is the lattice parameter. It should be noted that the pure translational group is a special case of the helical group: in this case the orthogonal transformation *R* is the identity operation, *i.e.* *Q* = 1 and *f* = *a*. The individual monomer has its own symmetry group, referred to as **P**, which belongs to one of the following axial point group families: **C**_*n*_, **S**_2*n*_, **C**_*nh*_, **D**_*n*_, **C**_*nv*_, **D**_*nd*_, **D**_*nh*_, with *n* = 1, 2, 3,…. If *n* = ∞, the corresponding LGs describe the symmetry of linear (strictly one-dimensional) systems. As **P** should preserve the *z* axis, but not necessarily its orientation, it is in many cases only a subgroup of the full symmetry group of the monomer.

The elements of LGs are combinations or products of the symmetries resulting from the arrangement of the monomers, described by an infinite Abelian group **Z**, and the internal symmetries of the monomer itself, described by an axial point group **P**. Therefore, the structural factorization of LGs can be expressed as **L** = **P** **Z** (direct or semi-direct product). There are infinitely many LGs which can be classified into 13 LG families, each having an infinite number of members. The factorization of LGs into a weak direct product of cyclic groups (Damnjanović & Vujičić, 1982 ▸) played a crucial role in proving the Jahn–Teller theorem for polymers and other quasi-1D crystals (Milošević & Damnjanović, 1993 ▸). It was used to find all conformation classes (orbit types) of the LG action, and further to determine the minimal sets of orbits that are sufficient to obtain a particular group of symmetry.

LGs also include symmetries of incommensurate structures, with helical periodicity only. Since they are not subgroups of the space groups like rod groups (see *IT* E), LGs have, thus far, been beyond the scope of classical crystallography. Specifically, due to the mono-periodicity, the crystallographic restrictions on the order *q* of the rotational axis imposed on the space (and layer) groups do not apply to the LGs. Therefore only 75 LGs are subgroups of the space groups. The order of the principal axis of rotation of the isogonal point group **P** of rod groups takes one of the crystallographic values *q* = 1, 2, 3, 4, 6 (or *q* = 1, 2, 3 for the groups with roto-reflections).

For some physical processes, Raman scattering for instance, the translational symmetry is irrelevant and only the orthogonal parts of the LG elements affect the process. Such orthogonal transformations form the isogonal (point) group **P**_I_. The axial point group **P** is a subgroup of **P**_I_. This means that the elements of **P**_I_ that are not the elements of **P** are not the symmetries of the considered system. Note that if an LG is incommensurate its isogonal group is infinite. In the case of the commensurate LGs, the translational group **T** is an invariant (normal) subgroup of **L**, and the isogonal point group is the factor group **P**_I_ = **L**/**T**. This means that the LG can be decomposed into the cosets **L** = **T** + (*R*_2_ ∣ *f*_2_)**T** + ⋯;. If **Z** = **T** all the coset representatives are found from **P** (all the fractional translations *f*_*i*_ vanish), and **P**_I_ = **P**. Such LGs are symmorphic. On the other hand, when **P** is a nontrivial subgroup of **P**_I_, **L** is a nonsymmorphic group.

The parameter *Q* of the helical group **T**_*Q*_(*f*) can be any real number, but it is only for the rational values *Q* = *q*/*r* (where *q* and *r* are positive co-prime integers; by convention *r* ≤ *q*) that there exists an index-*q* translational subgroup with the period *a* = *qf*. Such LGs and the corresponding configurations are called commensurate. In the case of incommensurate LGs, the parameter *Q* takes an irrational value, and such groups describe quasi-1D systems with helical periodicity only (without any translational symmetry).

For commensurate systems, together with monomers (minimal building blocks arranged by generalized translations **Z**), a unit cell can be introduced (an elementary cell related to the pure translations **T**). Furthermore, for both incommensurate and commensurate systems, it is of both technical and fundamental importance to introduce the concept of a symmetry cell, referred to as a symmcell (Damnjanović & Milošević, 2015 ▸). The symmcell is composed of a set of orbit representatives, and the entire system is derived from it through application of the full LG symmetry.

Whenever there is a helical symmetry, apart from linear quantum numbers of quasi-momentum *k*, taking values from the linear Brillouin zone (−π/*a*, π/*a*], and quasi-angular momentum *m*, taking integer values from the interval (−*n*/2, *n*/2] (also referred to as roto-translational quantum numbers), it is convenient to use helical quantum numbers 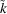 and 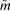 , which are canonically conjugated to the helix (instead of to the translational direction). For instance, in the case of helical group **T**_*Q*_(*f*), 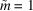 , as there are no pure rotations, while helical quasi-momentum 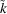 takes the values from the helical Brillouin zone (−π/*f*, π/*f*] which is (in the case of commensurate systems) *q* = *a*/*f* times enlarged relative to the linear Brillouin zone. On the other hand, in the case of incommensurate systems only the helical Brillouin zone is well defined.

Irreps of LGs can be induced starting from the one-dimensional irreps of the Abelian cyclic group **Z** using any of the two sets of quantum numbers, and by means of the transition rules (Damnjanović & Milošević, 2010 ▸) one can easily switch between the two representations. In the case of LGs with nontrivial helical symmetry only helical quantum numbers are conserved, while selection rules expressed in terms of the linear quantum numbers have nontrivial form. As stems from the LG factorization (Damnjanović & Vujičić, 1982 ▸), possible dimensions of LG irreps are 1, 2 and 4. Historically, the significance of commensurate LGs for polymers was recognized by Vainshtein (1959 ▸), who referred to them as ‘spiral groups’, and gave a comprehensive overview of crystallographic symmetry (Vainshtein, 1971 ▸) [see also Vainshtein (1994 ▸)]. Subsequently, LGs were constructed as extensions of one-dimensional translations by point groups (Vujičić, 1977 ▸), incorporating irrep induction through linear quantum numbers (Božović *et al.*, 1978 ▸).

Symmetries of chiral quasi-1D systems existing in two conformations which map to each other by spatial inversion (*e.g.* polymers exhibiting dichroism) are described by chiral LGs. These groups necessarily include a nontrivial helical axis, while spatial inversion cannot be their element. To enable a symmetry-based study of the optical activity of polymers and nanotubes, general matrix forms of the second-rank tensors for LGs have been derived (Milošević, 1995 ▸), and it has been shown that the materials invariant under infinite subsets of LGs have physical property tensors of the same form (Litvin, 2014 ▸).

### 3.1. Example: nanotubes

**Figure 4:**
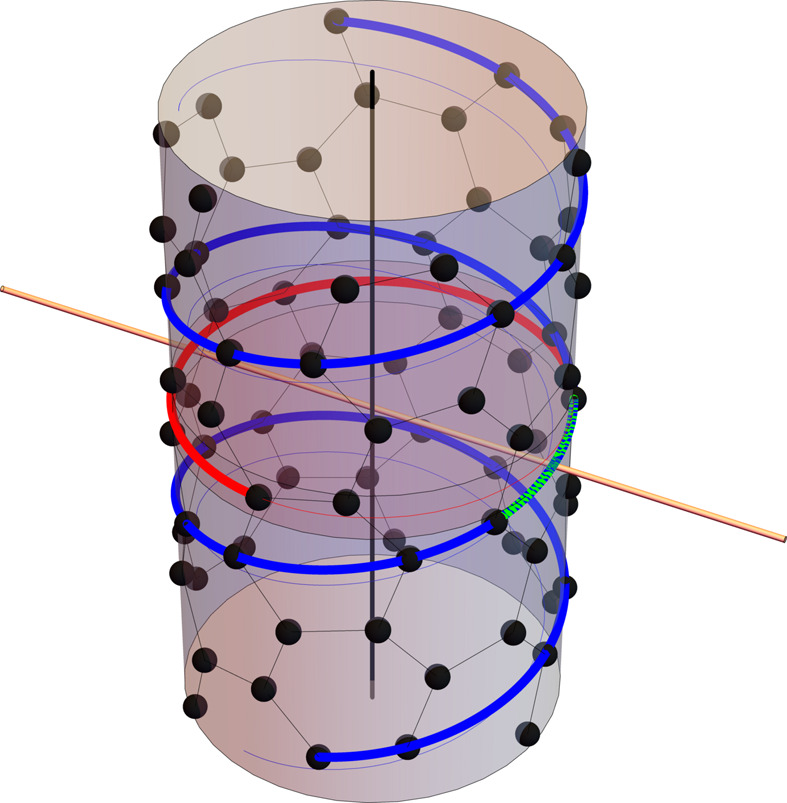
LG symmetry of a chiral single-wall CNT (6,3): **L**_(6,3)_ = **T**_42/23_ **D**_3_. The group generators: (i) 24π/21 rotation (around the nanotube axis) followed by a fractional translation *f* = 0.80 Å (along the nanotube axis), highlighted in blue; (ii) pure rotation (around the nanotube axis) for 2π/3, highlighted in red; (iii) π rotation around the (twofold) horizontal axis (perpendicular to the nanotube axis), highlighted in green. By successively applying the generators to an arbitrarily chosen carbon atom, the orbit representative C_0_, with cylindrical coordinates (3.11 Å, π/7, 0.23 Å), the entire nanotube is constructed (*i.e.* the symmcell consists of a single atom). On a different note, the (6,3) CNT is commensurate, with translational period *a* = 11.28 Å, and its unit cell comprises 84 atoms, while the monomer from which the nanotube can be constructed by the action of generalized translations **Z** = **T**_42/23_ (helical group) consists of six atoms. As **L**_(6,3)_ is a nonsymmorphic group, the isogonal point group **P**_I_ = **D**_42_ is not its subgroup and differs from the point factor **P** = **D**_3_.

Various properties of LGs can be illustrated on CNTs. Although single-wall CNTs appear in a myriad of different chiralities, all of them are single-orbital systems, and atoms of single-wall CNTs bijectively correspond to the elements of the LG which describes their symmetry. This means that by the LG action on an arbitrarily chosen carbon atom (the orbit representative C_0_ producing the unit element of the group) the entire single-wall CNT is built (Fig. 4 ▸).

Single-wall CNTs of nontrivial chiralities (*n*_1_, *n*_2_) and (*n*_2_, *n*_1_), where *n*_1_ ≠ *n*_2_, are enantiomers, and their symmetry is described by helical groups of the fifth LG family, the groups lacking spatial inversion.

The vast majority of double-wall CNTs and other multi-wall CNTs do not have any translational symmetry, but do have a helical ordering, and the only possibility of applying the (generalized) Bloch theorem and all the underlying solid-state theory is based on the incommensurate LGs.

**Figure 5:**
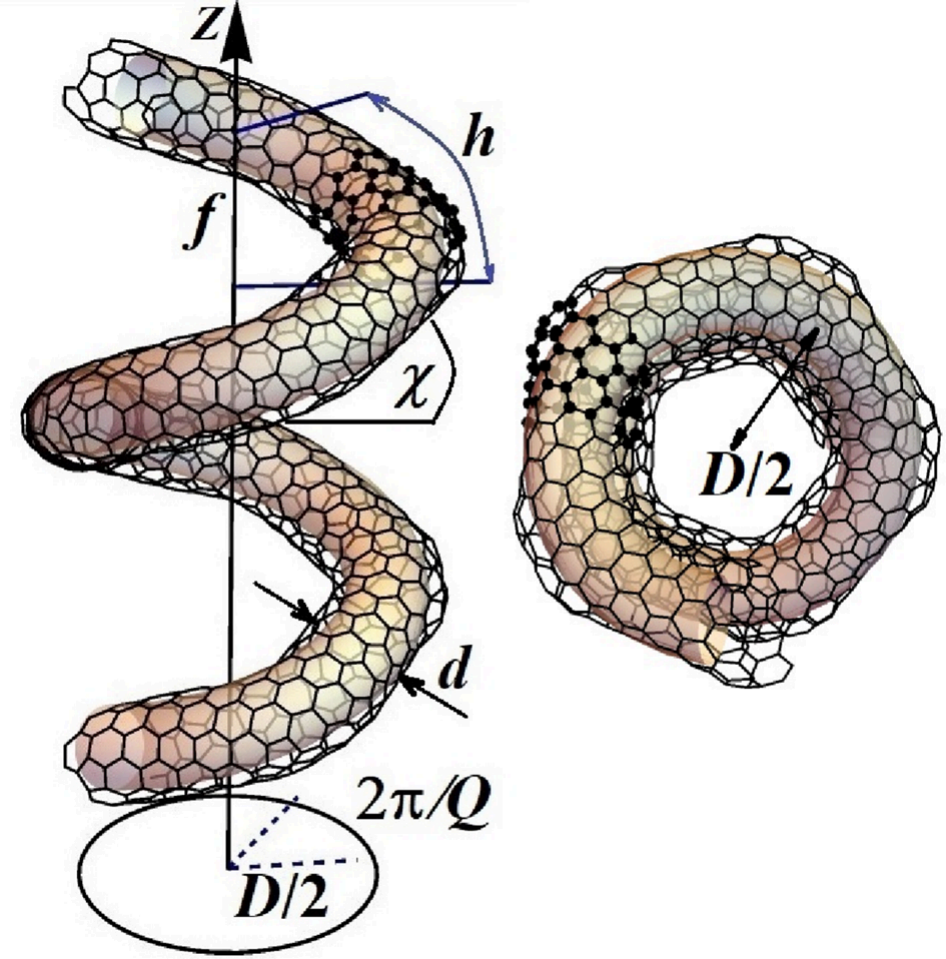
LG symmetry of helically coiled CNT (3,3,0,0,((1,0),(0,5))): **L**_HCCNT_ = **T**_19/4_ **D**_1_, generated by (i) 8π/19 rotation followed by a fractional translation *f* = 4.84 Å and (ii) π rotation around the axis perpendicular to the helical axis. Carbon atoms arrange themselves on a helical tubular surface, forming pentagons, hexagons and heptagons. The tube is commensurate, with translational period *a* = 91.87 Å. Since **L**_HCCNT_ is a nonsymmorphic group, the isogonal point group **P**_I_ = **D**_19_ is not its subgroup and differs from the point factor **P** = **D**_1_. The other structural parameters are as follows: helix diameter *D* = 21.98 Å, inclination angle of the helix χ = 19.42°, mean tubular diameter (as helically coiled CNTs are corrugated) *d* ≃ 7 Å, monomer length *h* = *f*/sinχ = 14.54 Å. The unit cell contains 2280 atoms, with the monomer comprising 120 atoms and the symmcell consisting of 60 atoms, highlighted in bold.

Moreover, there are structures like helically coiled CNTs (Popović *et al.*, 2014 ▸) for which, even when they have translational symmetry, application of the Bloch theorem does not lead to effective simplification and reduction of the calculations as the number of atoms in a unit cell is too large (Fig. 5 ▸). Therefore, in such cases it is essential to use the symmcell (rather than the unit cell) and to apply the generalized Bloch theorem which relies on the full LG (instead of on its translational subgroup).

In practical settings, LG symmetry has played a pivotal role in understanding and elucidating the qualitative change in conducting properties of CNTs when placed on a substrate (Petrov & Rotkin, 2003 ▸), and has contributed to understanding the modulation of the CNT electronic band structure in an external field (Li *et al.*, 2003 ▸), as well as enabling the design of an electromechanical switch based on pentaheptite nanotubes (Milošević *et al.*, 2007 ▸).

Finally, LGs are the symmetry groups of all the possible types of (either existing or hypothetical) nanotubes, rolled up from arbitrary two-dimensional lattices along an arbitrary chiral vector (Damnjanović *et al.*, 2007 ▸).

## 4. Conclusions

As demonstrated in this paper, numerous one-dimensional crystals cannot be described by either space or subperiodic groups (frieze, rod and layer groups), thus requiring the application of line groups. Currently, the use of these groups is not very common in crystallography, but they are essential to describe these types of systems. The diversity of line groups far exceeds that of space groups and stands out as a defining feature, emphasizing the broad spectrum of properties showcased by quasi-1D crystals. There are cases in which these crystals can be described by one of the 75 crystallographic rod groups, which are also line groups. The correct characterization of the symmetry of these one-dimensional crystals is necessary for the study of these materials which, due to their unique properties, hold significant importance in various fields like nanotechnology and nanomedicine, biotechnology, and pharmacy. Because of the constrained structure, small size, high aspect ratio and unique surface properties, applications of quasi-1D compounds offer tailored solutions to various challenges in these fields, by enabling precise control and manipulation at a cellular or molecular level.
